# Anti-oxidant effect of nitrite in the pancreatic islets of type 2 diabetic male rats 

**DOI:** 10.22038/IJBMS.2023.68245.14900

**Published:** 2023-04

**Authors:** Asghar Ghasemi, Sevda Gheibi, Khosrow Kashfi, Sajad Jeddi

**Affiliations:** 1 Endocrine Physiology Research Center, Research Institute for Endocrine Sciences, Shahid Beheshti University of Medical Sciences, Tehran, Iran; 2 Department of Clinical Sciences in Malmö, Unit of Molecular Metabolism, Lund University Diabetes Center, Clinical Research Center, Lund University, Malmö, Sweden; 3 Department of Molecular, Cellular and Biomedical Sciences, Sophie Davis School of Biomedical Education, City University of New York School of Medicine, New York, USA

**Keywords:** Nitric oxide, Nitrite, Oxidative stress, Pancreatic islets, Rat, Type 2 diabetes

## Abstract

**Objective(s):**

Nitrite, a nitric oxide (NO) donor, increases insulin secretion from pancreatic islets and has positive metabolic effects in type 2 diabetes (T2D). Here, we test the hypothesis of whether nitrite-induced insulin secretion is due to blunting of diabetes-induced oxidative stress in the islets.

**Materials and Methods:**

T2D was created in male rats using a combination of streptozotocin at 25 mg/kg and a high-fat diet. Wistar rats were assigned to 3 groups (n=6 in each group), including control, T2D, and T2D+nitrite; the latter group consumed drinking water containing sodium nitrite (50 mg/l) for eight weeks. At the end of the study, mRNA levels of NADPH oxidase (Nox1, 2, 3, and 4), superoxide dismutase (SOD1, 2, and 3), glutathione peroxides (GPX1 and 7), glutathione reductase (GR), catalase, thioredoxin (TXN1 and 2), and thioredoxin reductase (TXNRD1) were measured in the isolated pancreatic islets.

**Results:**

In the islets of diabetic rats, mRNA expressions of Nox1, 2, and 4 were higher, whereas expressions of SOD1, 2, catalase, GPX1, 7, GR, and TXN1 were lower than controls. Nitrite significantly (all *P*-values<0.05) decreased gene expression of Nox1 (0.39-fold) and Nox4 (0.23-fold) and increased gene expression of SOD1 (2.2-fold), SOD2 (2.8-fold), catalase (2.7-fold), GPX1 (2.2-fold), GPX7 (6.0-fold), GR (3.0-fold), TXN1 (2.1-fold), and TXNRD1 (2.3-fold) in diabetic rats.

**Conclusion:**

Nitrite decreased oxidative stress in isolated pancreatic islets of rats with T2D by suppressing oxidants and augmenting anti-oxidants. These findings favor the notion that nitrite-induced insulin secretion is partially due to decreased oxidative stress.

## Introduction

Diabetes is linked with an astonishing death rate of one person every eight seconds (1). Therefore, type 2 diabetes (T2D) management needs to change from only a glycemic-based to a pathophysiological-based view (2). In addition, T2D is related to reduced bioavailability of nitric oxide (NO) (3); thus, a novel strategy for the management of T2D is to increase NO bioavailability (4). 

Experimental studies have displayed that nitrite, an NO donor, has favorable metabolic effects in T2D via increasing insulin secretion from the pancreas and decreasing insulin resistance (IR) (5-7). Nitrite improves IR in T2D by multiple mechanisms, including *(i)* enhancing glucose uptake in the adipose tissue (8); *(ii)* insulin-independent movement of glucose transporter 4 (GLUT4) to the membrane (6) via sirtuin3-AMP-activated protein kinase (AMPK)-dependent pathway (9) in skeletal muscle cells; *(iii)* decreasing adipocyte size (5); *(iv)* decreasing transcription of cytokines involved in inflammation in the adipose tissue (5); and *(v)* browning of white adipose tissue (WAT) (10). Nitrite stimulates insulin secretion by increasing pancreatic islet insulin content (7) and blood flow (11). Moreover, we recently reported that nitrite administration potentiates pancreatic insulin secretion via increasing insulin exocytosis (12). 

Oxidative stress is related to the onset and development of T2D (13). Pancreatic β-cells constitutively have a weak anti-oxidant defense system (14-16) and are at high risk of oxidative impairment (17). The sensitivity of rat islets to peroxide radicals is 25 times higher than the liver (18). The anti-oxidant protective capacity of the pancreatic β-cells may not be sufficient to deal with the challenges of modern lifestyles (14). Under a hyperglycemic environment in T2D, reactive oxygen species (ROS) are formed in pancreatic islets (19). This can impair insulin secretion (17, 20) and contribute to β-cell loss and function (16). Nitrite has anti-oxidant properties in the heart and vascular tissue (21-23), as well as in lipopolysaccharide (LPS)-activated mouse macrophages and human monocytes (24). Favorable metabolic effects of nitrite and its stimulatory effects on insulin secretion, synthesis, and exocytosis from pancreatic β-cells in T2D rats have been reported (7, 11, 12); however, the underlying mechanisms of nitrite-induced insulin secretion have not been fully elucidated. Oxidative stress plays a key role in decreased insulin secretion; therefore, we asked the question as to whether nitrite’s stimulatory effect on glucose-induced insulin secretion (GIIS) in T2D is mediated by reducing oxidative stress in the pancreatic islets. We thus measured mRNA levels of the genes that play a key role in islets’ oxidative stress; for this purpose, we used isolated pancreatic islets from diabetic rats. 

## Materials and Methods

***Animals***

Male Wistar rats (n=18, 2-month-old, 190–210 g) were kept in regular conditions with water and food *ad libitum*. The ethics committee of the Research Institute for Endocrine Sciences affiliated with Shahid Beheshti University of Medical Sciences approved all experimental procedures of the current study (IR.SBMU.ENDOCRINE.REC.1400.055; approved date: 2021-08-15).

***Timeline of the study***

**Figure 1:**
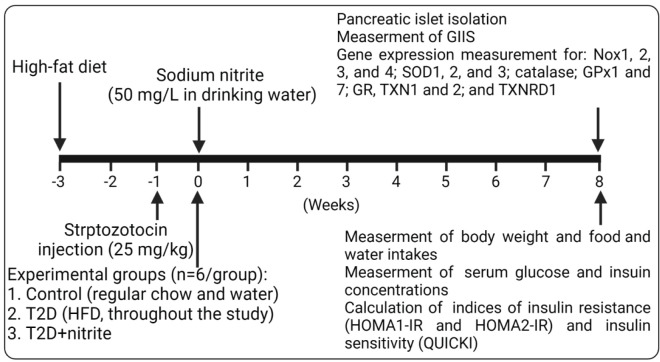
Study timeline

T2D: Type 2 diabetes; HOMA1-IR: Homeostasis model assessment of insulin resistance (IR); HOMA2-IR: Updated HOMA-IR; GIIS: Glucose-induced insulin secretion; QUICKI: Quantitative insulin-sensitivity check index; GPx: Glutathione peroxidase; SOD: Superoxide dismutase; TXN: Thioredoxin; TXNRD: Thioredoxin reductase; Nox: Nicotinamide adenine dinucleotide phosphate (NADPH) oxidase ; HFD: High-fat diet

This study is an interventional experimental study**;** Figure 1 presents the timeline of the study. Rats were allocated to 3 groups (n=6 in each group), including control, T2D, and T2D+nitrite. Animals in the T2D+nitrite group consumed drinking water containing sodium nitrite at 50 mg/L for 8 weeks, whereas rats in other groups received tap water only. This nitrite dose was chosen based on previous reports from our laboratory (7) and others (6, 25, 26), showing it to be safe, and producing favorable metabolic effects.

To measure water consumption (ml/24 hr/rat) and food intake (g/24 hr/rat), a certain amount of water (1000 ml in 2 water bottles) and food (500 g) was added to each cage (3 rats/cage). Then, the water remaining in the water bottles and food remaining in the cages at the end of each week were registered for 8 successive weeks using a graduated cylinder and a digital scale, respectively. In addition, levels of glucose and insulin were measured in the serum of fasted rats, and indices of IR [Homeostasis Model Assessment of IR (HOMA1-IR), updated HOMA-IR (HOMA2-IR)], and insulin sensitivity (quantitative insulin-sensitivity check index (QUICKI) were calculated as described previously (27). Overnight (12 hr) fasted rats were anesthetized with sodium pentobarbital (60 mg/kg), and blood samples were collected by cutting the tail tips at week 0 (for the confirmation of diabetes in rats, which was concurrent with the start of nitrite administration) and week 8 (end of the nitrite administration). At week 8, pancreatic islets from all rats were separated by the Lacy & Kostianovsky method (7), GIIS, and mRNA of nicotinamide adenine dinucleotide phosphate (NADPH) oxidase isoforms (Nox1, 2, 3, and 4), superoxide dismutase isoforms (SOD1, 2, and 3), glutathione peroxidase (GPX1 and 7), glutathione reductase (GR), catalase, thioredoxin (TXN1 and 2), and thioredoxin reductase (TXNRD1) were measured in isolated pancreatic islets using real-time PCR.

***Induction of T2D***

T2D was induced in rats using a high-fat diet (HFD) and streptozotocin (STZ) (28). Briefly, after 14 days of HFD feeding (29, 30), 25 mg/kg of STZ was intraperitoneally injected in all fasted rats in diabetic groups, and 7 days later, male rats with fasting serum glucose higher than or equal to 150 mg/dl were allocated in T2D groups, which continued to receive the HFD for the rest of the study (28).

For the preparation of the HFD, 1000 g of powdered regular pellet diet (Pars animal feed Company, Tehran, Iran), 531 g sheep butter as a source of fat, 125 g of casein (Iran Caseinate Company, Karaj, Iran) as a source of protein, 3 g of DL-methionine (Behparvar Company) to overcome low sulfur amino acids in the casein, 7 g of vitamin mix (Behroshd Company, Saveh, Iran), and 42 g mineral mix (Behroshd Company, Saveh, Iran) were thoroughly mixed to produce 1708 g of HFD. In the regular pellet diet, the total caloric value was ∼3160 kcal/kg, and calories received from fat, carbohydrate, and protein were 5.7, 72.2, and 22.1%, respectively (Table 1). In the prepared HFD, the total caloric value was ∼4900 kcal/kg, and calories received from fat, carbohydrate, and protein were 58.8, 27.0, and 14.2%, respectively. In the HFD/low-dose STZ model of T2D, rats consume HFD to induce IR, and after that, low-dose of STZ causes partial destruction of pancreatic β-cells. STZ dose (15–40 mg/kg), percent of calories received from fat (30–67%), and duration of HFD before STZ injection (14–84 days) vary between studies (28). However, most studies, including the initial ones that introduced the model (29, 30), used 14 days on HFD before STZ injection, as we did in the current study. 

***Serum glucose and insulin measurement***

Fasting serum glucose was measured using the glucose oxidase method (Pars Azmoon, Tehran, Iran, Cat. No.97001). Fasting serum insulin was measured using a rat-specific ELISA kit (Mercodia, Sweden, Cat. No.101113-10), respectively. The intra-assay coefficient of variations for glucose and insulin were 1.4% and 8.4%, respectively.

***Expression of target genes***

The sequence of primers is shown in Table 2. Total RNA from isolated pancreatic islets was extracted by the RNX-Plus solution kit (Cinagen Co., Tehran, Iran, Cat. No. EX6101). A NanoDrop-1000 spectrophotometer (Thermo Scientific, USA) was used to determine purity and quantity of the extracted total RNA. For the cDNA synthesis, extracted RNA was reverse transcribed with a cDNA synthesis kit (SMOBiO Technology, Taiwan). Related products in this kit are Reverse transcriptase (Cat. No.RP13002108402-1), RNase inhibitor (Cat. No.RL10002108111-8), dNTP Mix (Cat. No.CD10102108400-1), Oligo (dT) (Cat. No.CHRP032208402-1), Random Hexamers, (Cat. No.CHRP0342106600-1), and DEPC-Ttrated H_2_O (Cat. No.CHRP0521081101). Finally, 1 μl cDNA was amplificated in a real-time PCR machine (Rotor-Gene 6000, Corbett, Life science, Sydney, Australia) by a SYBR Green Master Mix (Thermo Fisher, USA Cat. No. 4309155). PCR reaction contained 1 µl cDNA, 1 μl of primers (forward and reverse), 7.5 μl Master Mix, and 5.5 μl DEPC-treated water, yielding a total volume of 15 µl. The thermal cycling settings included a 10 min (95 °C) initial denaturation followed by 40 cycles with 45, 45, and 60 sec at 94, 58, and 72 °C, respectively, and a five-min final extension at 72 °C. 

***Statistical analyses***

Statistical analysis was performed by GraphPad Prism software; all values are presented as mean±SEM. One-way analysis of variance followed by the Tukey *post hoc* test was used to compare water consumption and food intake, body weight, serum glucose and insulin, indices of insulin sensitivity/resistance, and insulin secretion at week 8. Relative expressions of genes were calculated based on their cycle thresholds versus β-actin as a reference gene with the REST software (31). This software uses a randomization test to compare the difference between control and treated samples, which avoids any assumptions about data distribution and is therefore preferred over parametric tests (31). The number of randomizations was set at 2000, which provides a reliable estimate of the *P*-value<0.05 (31). 

## Results

***Verification of model***

Consumption of HFD for two weeks before STZ injection produced IR in rats as indicated by higher HOMA1-IR (7.60±0.41 vs 2.86±0.27, *P*<0.001) and HOMA2-IR (3.86±0.21 vs 1.88±0.24, *P*<0.01) and lower QUICKI (0.287±0.002 vs 0.331±0.006, *P*<0.001) compared with controls. The success rate for induction of the T2D model was 63% (12/19); the range of glucose and insulin concentrations in the diabetic rats were 161–208 mg/dl and 113-181 pmol/L, respectively. At the end of the study, higher serum glucose (101%, *P*<0.001), serum insulin (124%, *P*<0.001), and lower GIIS from isolated islets (45%, *P*<0.001) were detected in diabetic rats. Moreover, rats with T2D had a higher HOMA1-IR (334%) and HOMA2-IR (167%), and lower QUICKI (17.6%) at the end of the study.

***Effect of nitrite on final body weight and intakes of water and food***

Rats with T2D had higher body weight by 12.7% (*P*<0.001), and water consumption by 46% (*P*<0.001) at week 8 while they had lower food intake by 27% (*P*<0.001) than controls. As shown in Table 3, nitrite administration in rats with T2D decreased body weight by 8.6% (*P*<0.001) but had no significant effect on water and food intake at week 8. 

***Effect of nitrite on serum glucose and insulin, insulin sensitivity/resistance, and insulin secretion***

As shown in Table 4, nitrite administration for 8 weeks to the rats with T2D decreased the concentration of serum glucose by 17.3% (*P*<0.01) and serum insulin by 19.6% (*P*<0.05), and increased islets GIIS by 39.1% (*P*<0.001). In addition, nitrite administration for 8 weeks to the rats with T2D resulted in decreased HOMA1-IR by 34.3% (*P<*0.001) and HOMA2-IR by 25.0% (*P*<0.01) and increased QUICKI by 7.5% (*P*<0.05). 

***Effect of nitrite administration on oxidative stress-related genes in islets of rats with T2D***

**Figure 2:**
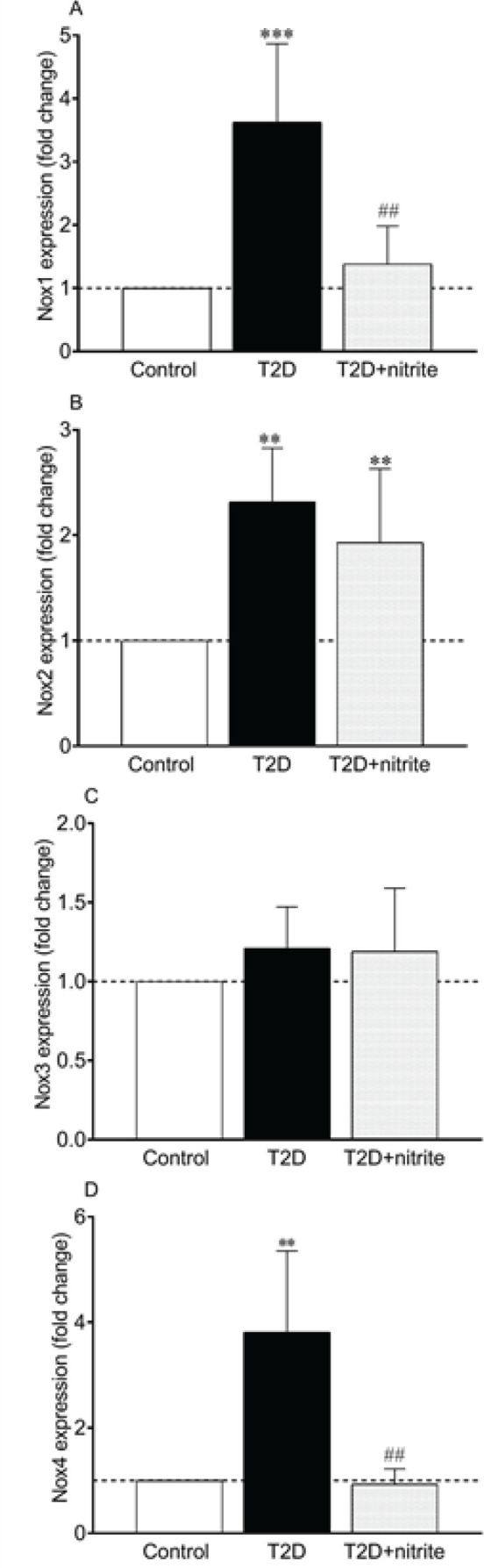
Effect of nitrite administration on mRNA expression of NADPH oxidase (Nox) isoforms [Nox1 (A), Nox2 (B), Nox3 (C), and Nox4 (D)] in rats with type 2 diabetes (T2D). Symbols ** and *** show significant differences at *P*<0.01 and *P*<0.001, respectively, vs the control group. Symbol ## shows a significant difference at *P*<0.01 vs the diabetic group. Values are mean±SEM (n=6/group)

As shown in Figure 2, mRNA levels of Nox1, 2, and 4 were significantly higher in isolated islets of diabetic rats by 3.6, 2.3, and 3.8 folds, respectively; however, no change was observed in mRNA expression of Nox3 (Figure 2C). In addition, compared with the islets from T2D rats, nitrite administration decreased Nox1 (0.39-fold, *P*<0.01) and Nox4 (0.23-fold, *P*<0.01) gene expression (Figures 2A and 2D) in the diabetic rats. But, it had no significant effect on Nox2 and Nox3 expression (Figures 2B and 2C). Higher expression of Nox2 (1.9-fold. *P*<0.0**5**) was also observed in the T2D+nitrite group compared with the control group. 

**Figure 3:**
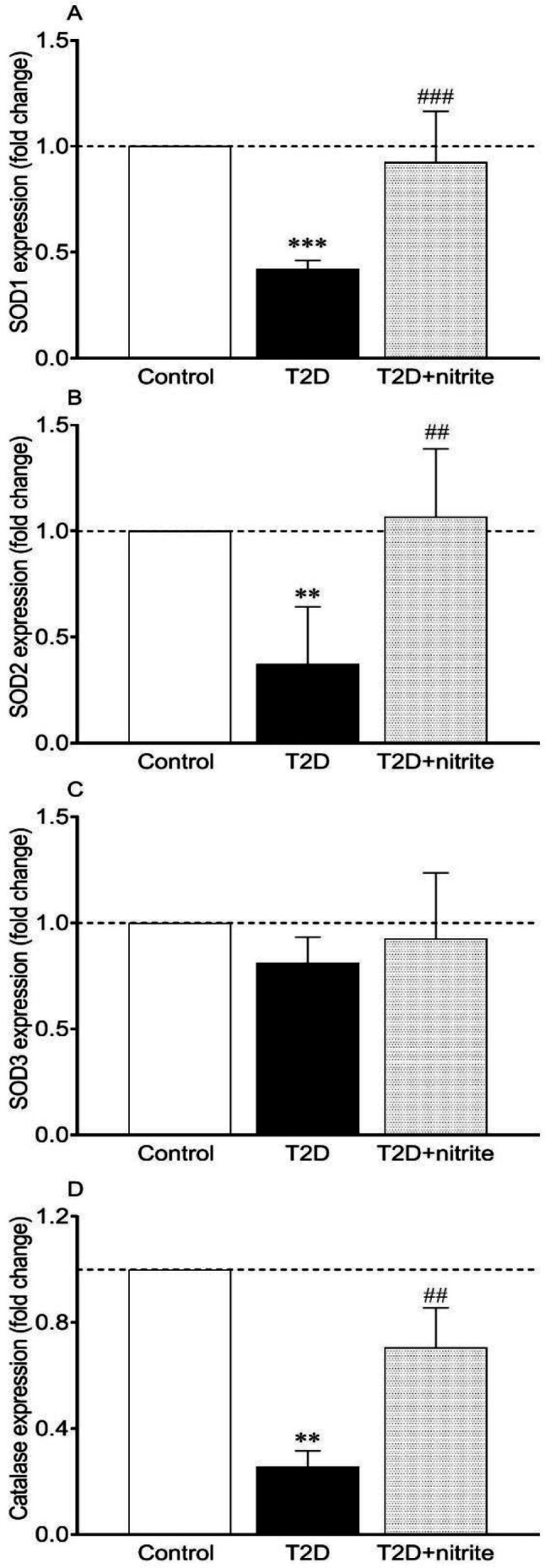
Effect of nitrite administration on mRNA expression of superoxide dismutase (SOD) isoforms [SOD1 (A), SOD2 (B), and SOD3 (C)] and catalase (D) in rats with type 2 diabetes (T2D). Symbols ** and *** show significant differences at *P*<0.01 and *P*<0.001, respectively, vs the control group. Symbols ## and ### show significant differences at *P*<0.01 and *P*<0.001, respectively, vs the diabetic group. Values are mean±SEM (n=6/group)

As shown in Figure 3, expressions of SOD1 and 2, and catalase were significantly lower in isolated islets of diabetic rats by 0.42, 0.37, and 0.26 folds, respectively. mRNA expression of SOD3 was comparable between groups (Figure 3C). Moreover, compared with non-treated diabetic rats, administration of nitrite to the diabetic rats increased mRNA expressions of SOD1 (2.2-fold, *P*<0.001), SOD2 (2.8-fold, *P*<0.01), and catalase (2.7-fold, *P*<0.01) without affecting SOD3 expression (Figure 3C). 

**Figure 4:**
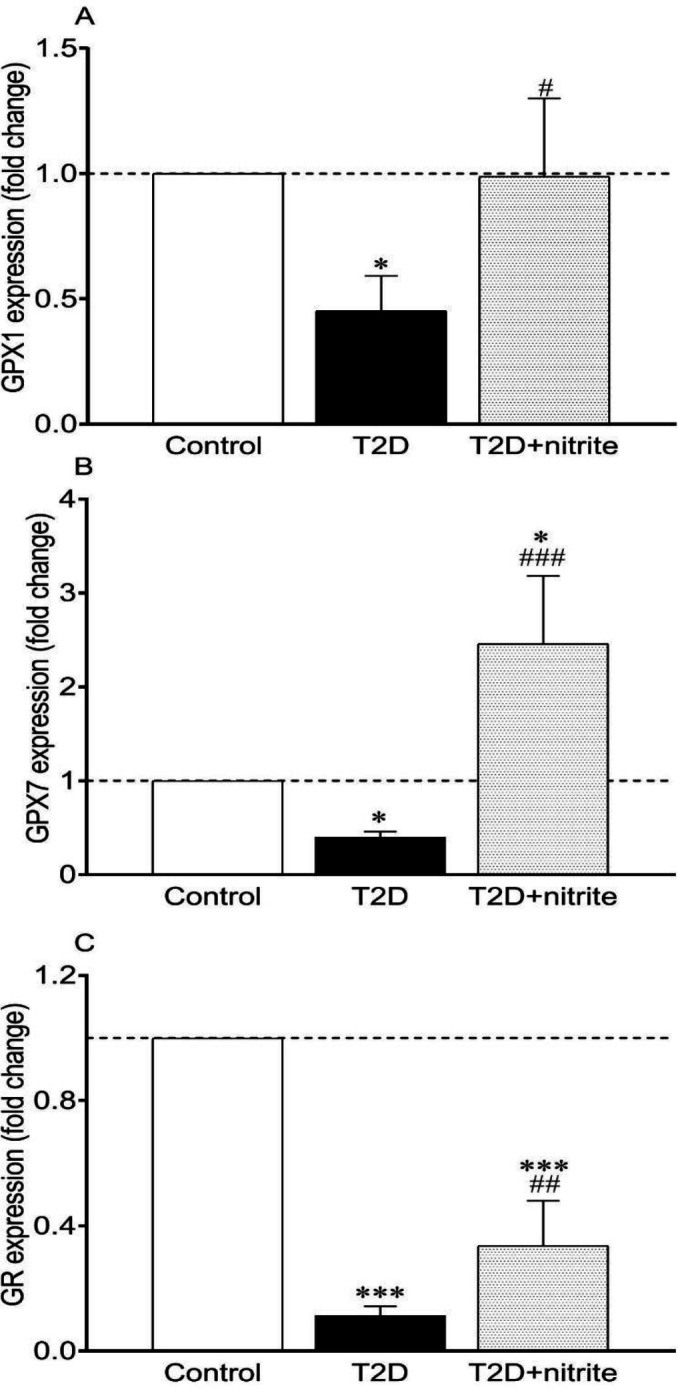
Effect of nitrite administration on mRNA expression of glutathione peroxidase (GPX) isoforms [GPX1 (A) and GPX7 (B)] and glutathione reductase (GR, C), in rats with Type 2 diabetes (T2D). Symbols * and *** show significant differences at *P*<0.05 and *P*<0.001, respectively, vs the control group. Symbols #, ##, and ### show significant differences at *P*<0.05, *P*<0.01, and *P*<0.001, respectively, vs the diabetic group. Values are mean±SEM (n=6/group)

As shown in Figure 4, compared with controls, the following mRNA expressions were lower in isolated islets of rats with T2D: GPX1 (0.45-fold, *P*<0.05), GPX7 (0.40-fold, *P*<0.05), and GR (0.11-fold, *P*<0.001). Nitrite administration increased the expression of the following genes: GPX1 (2.2-fold, *P*<0.05), GPX7 (6.0-fold, *P*<0.001), and GR (3.0-fold, *P*<0.01). Higher expression of GPX7 (2.5-fold, *P*<0.05) and lower expression of GR (0.34-fold, *P*<0.001) were also observed in the T2D+nitrite group in comparison with the control group. 

**Figure 5:**
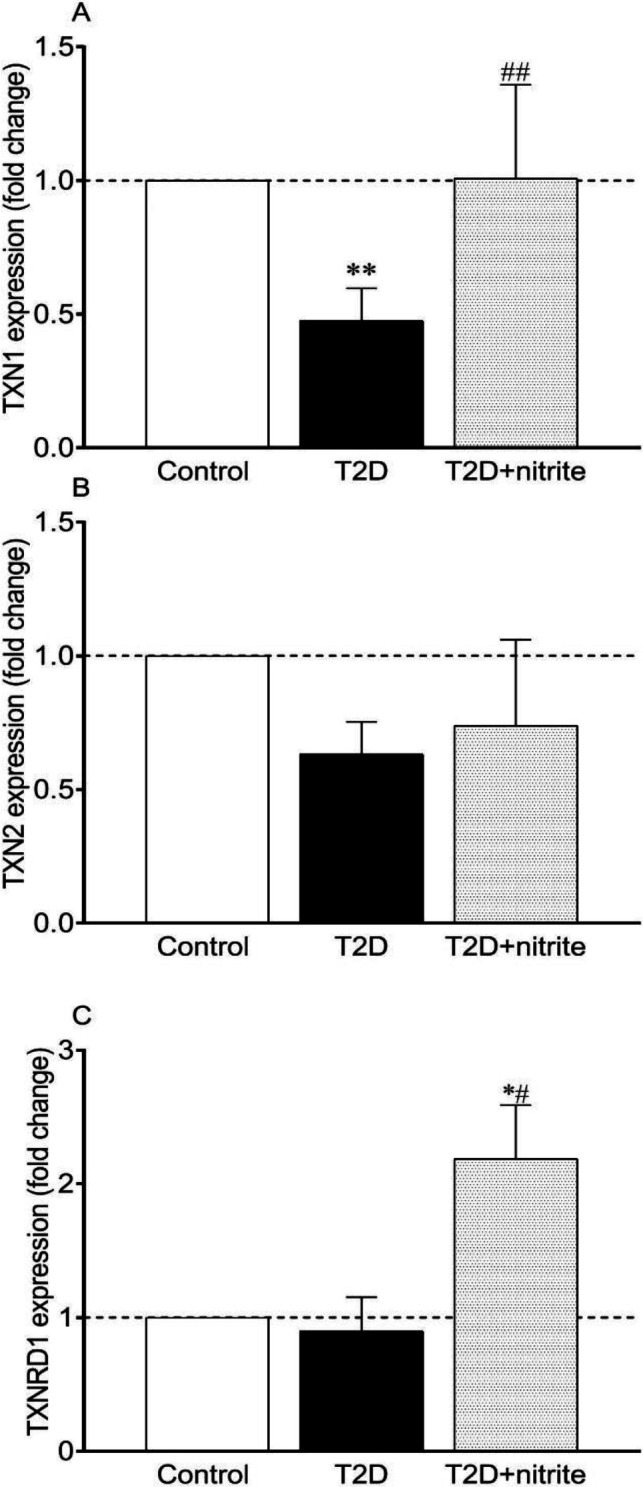
Effect of nitrite administration on mRNA expression of thioredoxin (TXN) isoforms [TXN1 (A) and TXN2 (C)], and thioredoxin reductase (TXNRD1, C), in rats with Type 2 diabetes (T2D). Symbols * and ** show significant differences at *P*<0.05 and *P*<0.001, respectively, vs the control group. Symbols # and ### show significant differences at *P*<0.05 and *P*<0.001, respectively, vs the diabetic group. Values are mean±SEM (n=6/group)

As shown in Figure 5, the expression of TXN1 was lower in isolated islets of diabetic rats by 0.48 **(***P*<0.0**1)**; however, no change was observed in the mRNA expression of TXN2 and TXNRD1 (Figure 5B and 5C). Moreover, administration of nitrite to the diabetic rats increased mRNA expressions of TXN1 (2.1-fold, *P*<0.01) and TXNRD1 (2.3-fold, *P*<0.05) in pancreatic islets of T2D rats, but it did not affect the expression of TXN2 (Figure 5C). Higher expressions of TXNRD1 (2.2-fold, *P*<0.05) were also observed in the T2D+nitrite group compared with the control group. 

## Discussion

The main result of the current study is that nitrite therapy attenuates T2D-induced increases in oxidative stress in isolated rat pancreatic islets. This finding indicates that the nitrite-induced increase in insulin secretion reported in our previous study (7) is at least in part due to blunting diabetes-induced oxidative stress in pancreatic islets. 

In this study, serum glucose, serum insulin, and HOMA-IR were higher, and GIIS was lower in diabetic rats, indicating increased IR and reduced insulin secretion in the HFD-STZ model of T2D. Over an 8-week treatment by nitrite in diabetic rats, serum glucose and insulin concentrations were reduced, accompanied by decreased IR and increased GIIS; these findings are similar to others (5-7, 12). The positive effect of nitrate/nitrite on glucose homeostasis in rats with T2D is due to reduced IR, increased insulin secretion, and pancreatic islet blood flow (11), increased glucose uptake in the skeletal muscle (6), increased expression of NO synthase enzymes in tissues sensitive to insulin effects (32), and increased insulin exocytosis (12). In addition, nitrite decreased body weight without affecting food intake in diabetic rats, suggesting that the weight-reducing effect of nitrite was not associated with food intake in this study. The body weight-reducing effect of nitrate/nitrite in diabetic rats is due to WAT browning (10, 33). 

In our study, mRNA levels of Nox1, 2, and 4 but not Nox3 were higher in the pancreatic islets of diabetic rats. Nitrite restored elevated expression of Nox1 and 4 but did not affect Nox2 and 3. The mitochondria, peroxisomes, endoplasmic reticulum, and cytosol are the primary sources that produce ROS in the pancreatic β-cells (14, 34). Isolated rat islets express mRNAs of Nox1, 2, and 4 in a constitutive manner (35), where Nox1 and 2 are co-localized in the cell membrane and Nox4 is expressed in the ER, mitochondria, cell membrane, and the pancreatic β-cell nucleus (34). In line with our results, expression of Nox (subunit p22^phox^, which is critical for Nox1-4 function (36)) is higher in the islets isolated from patients with T2D (37). In addition, increased mRNA levels of Nox1, 2, and 4 but not Nox3 participate in the metabolic dysregulation of β-cells (36), whereas inhibition of Nox4 protects human islets against glucolipotoxicity (38). Decreased activity or expression of Nox enzymes are among the possible mechanisms by which nitrite/nitrate can potentially reduce oxidative stress (39). Nitrite inhibits Nox activity in the kidney (39), liver (40), macrophages (24), and vascular tissue (21). Nitrite-induced decreased Nox gene expression, as observed in our study for Nox1 and Nox4 in the pancreatic islets, has been reported for Nox subunit p67 protein (part of in Nox2 and 3 holoenzymes) in the aorta (26) but not in the kidney (39). This differential expression suggests that nitrite may exert its effects on Nox isoforms by different mechanisms in different tissues. 

We showed that mRNA levels of SOD1 and SOD2 but not that of SOD3 were lower in the isolated islets of rats with T2D; nitrite increased SOD1 and SOD2 expressions to their natural values but did not affect SOD3 expression. In the pancreatic β-cells, Cu-Zn-SOD1 is expressed in the mitochondrial intermembrane space, cytosol, peroxisome, and the nucleus (34). Mitochondrial Mn-SOD2 and Cu/Zn-SOD3 (EC-SOD) target the mitochondrial matrix and extracellular space, respectively (34). Compared with the liver, SOD1 and SOD2 mRNA expressions in the pancreatic islets of male Wistar rats have been reported to be lower by 23% and 55%, respectively (41). In line with our results, lower expression of SOD1 (37, 42) and 2 (37) were observed in the pancreatic islets of patients with T2D (42). Nitrite administration (50 mg/L) in drinking water for three weeks restores the decreased SOD activity in the aorta of aged (26 to 28-month-old) mice, but it did not affect SOD1 and SOD2 protein expression (26). However, nitrite administration did increase SOD1 mRNA expression in the mesenteric arteries of hypertensive rats (23) and cardiac tissue of mice with congestive heart failure (CHF) (43).

In the current study, rats with T2D had lower islet expression of catalase, GPX (GPX1 and 7), and GR. Nitrite increased the transcription of these genes, particularly that of GPX7. Catalase and cytoplasmic GPX are hydrogen peroxide-inactivating enzymes (41), of which very low levels are found in the pancreatic islets (41). Catalase and GPX activities in the pancreatic rat islets are 1-3.5% (18, 41) and 21% (41) of those found in the liver. Catalase and GPX1 localize in the peroxisomes and cytosol, whereas GPX7 localizes in ER (34). Thus, overexpression of GPX and catalase protect the β-cells against hydrogen peroxide toxicity (41). In addition, GPX1 overexpression prevents, whereas buthionine sulfoximine, which depletes cellular glutathione levels, augments ribose-induced increases in peroxide levels, leading to decreased insulin mRNA, insulin content, and GIIS in rats islets (44). In line with our results, nitrite increases mRNA levels of catalase and GPX1 in vascular tissue from hypertensive rats (23) and heart tissue of CHF mice (43). In addition, in the heart, nitrite increases the reduced/oxidized-glutathione (GSH/GSSG) ratio in rats exposed to hypoxia (45). Of significance, the SOD-to-catalase ratio is higher in insulin-producing cells than in other tissue, favoring the accumulation of hydrogen peroxide (41). In our study, than controls, the ratio of SOD3-to-catalase was 3.17 in the diabetic group; this decreased to 1.31 in the diabetes+nitrite group reflecting a 2.4-fold change, which is more relevant in determining overall sensitivity to hydrogen peroxide toxicity (41).

In the current study, rats with T2D had lower expression of TXN1; nitrite administration increased this and that of TXNRD1. TXN-TXNRD1 plays a significant role in detoxifying hydrogen peroxide in the β-cells (46). In line with our results, nitrite increases TXN1 gene expression in the vascular tissue of hypertensive rats (23), and NO donors increase TXNRD1 expression in pancreatic islets (47). S-nitrosylation, the NO-dependent alteration of protein thiols to form S-nitrosothiols (SNO), is one of the principal ways that NO exerts its effects, including gene transcription (48). GSH, thioredoxin, and their related redox systems are involved in SNO reduction (48). TXNRD1 reduces oxidized TXN and keeps it active (48). In addition, TXN1 can function as a denitrosylase to protect soluble guanylyl cyclase (sGC) sensitivity to NO (49). 

Previously we reported that in T2D, nitrite increases islet insulin mRNA levels (12) and content (7), stimulates insulin secretion from islets (7), and increases mRNA expression of proteins involved in insulin exocytosis (12). Other studies on the islets or β-cell lines show that high glucose concentrations increase intracellular ROS production (20, 44, 50), inhibiting insulin secretion. Ribose, which produces ROS more robustly than glucose, inhibits GIIS and decreases insulin content and mRNA levels; N-acetylcysteine prevents these effects (44). Tert-butyl hydroperoxide, an agent which induces oxidative stress, lowers islet GSH content by 37% and decreases GIIS by 67% (18). In addition, GIIS was potentiated by 38% in Nox4-deficient islets (20), indicating stimulatory effects of Nox4 inhibition on insulin secretion, as observed in our study following nitrite administration. Collectively, hyperglycemia increases ROS production, and long-term ROS increase can cause dysfunction and death of β-cell (34). Therefore, the anti-oxidant effects of nitrite on the pancreatic β-cells and its effect on increasing insulin secretion are relevant because T2D is frequently accompanied by oxidative stress. Dietary consumption of green leafy vegetables would be a viable means of providing adequate nitrate/nitrite supplementation in T2D patients. Drugs targeting oxidative stress may be advantageous in the future treatment of T2D (34), and enhancing β-cell anti-oxidant activity may preserve residual β-cell function after the onset of T2D (44).

As for strengths, first, we provided efficiency-corrected relative gene expression, which is highly recommended (31) and prevents any miscalculated differences in expression ratios (51). Second, in the model of T2D in the current study, HFD induces IR, and STZ induces partial β-cell dysfunction; metabolic characteristics of this model, therefore, mimic the pathophysiology of T2D in humans (28). Finally, the dose of nitrite (50 mg/l in drinking water) used in the current study is ~5.8 mg/kg (based on 38 ml/day of water consumption and 330 g of average body weight in nitrite-treated rats), which translates to a human equivalent dose of 0.93 mg/kg (52). This nitrite dose is achievable through vegetable and fruit consumption, it is safe, and represents a low nitrite dose in humans (10, 53). As a limitation, the expression of the protein of studied genes was not measured in the current study. However, anti-oxidant enzyme activities of the tissues are chiefly determined by their mRNA levels (41), and expression of oxidative stress-response genes at mRNA levels in islets of rats with T2D is used as an indicator of β-cell oxidative stress (34, 54).

**Table 1:** Ingredient composition of regular diet and high-fat diet (28)

Ingredient	Regular diet		High-fat diet	
	Weight	Calorie	Weight	Calorie
Fat (%)	2	5.7	32.3	58.8
Carbohydrate (%)	57	72.2	33.4	27
Protein (%)	17.5	22.1	17.6	14.2
Vitamin and mineral mix (%)	4.9	-	5.7	-
Fiber (%)	6.6	-	3.9	-
Water (%)	12	-	7.0	-
Total calorie (kcal/100 g diet)	-	316	490	-

**Table 2:** Sequence of primers in Wistar rats

Name of primers	Accession number	Sequence of primers (5´→3´)	Product size
Nox1	NM_053683.2	F: TTCTAGAATAGCTACTGCCCACC R: AGGCTTTTTGCCAAAGTCGGAG	77
Nox2	NM_023965.1	F: CAGTGTGTCGGAATCTCCTCT R: ACCACTCCACGTTGAACAGA	168
Nox3	NM001004216.1	F: AGGTCGCATCATTCGAGGTC R: TTCGTCGAAGTGGTCTCTGC	79
Nox4	NM_053524.1	F: TAACCTCAACTGCAGCCTTATC R: CTTTTATCCAACAATCTCCTGGTTCTC	110
SOD1 (Cu/Zn SOD)	NM_017050.1	F: ATGGGGACAATACACAAGGC R: TCATCTTGTTTCTCGTGGAC	225
SOD2 (mitochondrial Mn-SOD)	NM_017051.2	F: AGGGCCTGTCCCATGATGTC R: AGAAACCCGTTTGCCTCTACTGAA	151
SOD3 (Cu/Zn extracellular SOD)	NM_012880	F: TCGAACTACTTTATGCCC R: GAAGACAAACGAGGTCTCTA	178
TXN1	NM_053800.3	F: CCTTCTTTCATTCCCTCTGTGA R: CCCAACCTTTTGACCCTTTTTA	143
TXN2	NM_053331.2	F: GAGACACCAGTTGTCGTGGA R: TGGCAAGGTCTGTGTGATCG	145
TXNRD1	NM_001351983.1	F: AAGGTGACCGCTAAGTCCAC R: CATTGATCTTCACGCCCACG	130
GPX1	NM_030826.3	F: CACAGTCCACCGTGTATGCC R: AAGTTGGGCTCGAACCCACC	292
GPX7	NM_001106673.1	F: CCTGCCTTCAAATACCTAACCC R: TGTAATACGGGGCTTGATCTCC	141
Catalase	AH004967.1	F: GTCCGATTCTCCACAGTCGC R: CGCTGAACAAGAAAGTAACCTG	272
ß-actin	NM_031144.3	F: CACCCGCGAGTACAACCTTC R: CCCATACCCACCATCACACC	207

Nox: NADPH oxidase; TXN: Thioredoxin; TXNRD: Thioredoxin reductase; GPX: Glutathione peroxidase; SOD: Superoxide dismutase

**Table 3:** Effects of 8-week nitrite administration on final body weight and intakes of water and food in rats with type 2 diabetes

**Parameters **	**Groups**		
	Control	Type 2 diabetes (T2D)	T2D +nitrite
Body weight (g)	321.0±3.1	362.0±5.4*	330.8±7.9#
Water consumption (ml/24 hr/rat)	25.8±0.9	37.5±0.6*	37.9±1.5*
Food intake (g/24 hr/rat)	18.7±2.4	13.7±0.1*	12.6±0.5*

*, # *P*<0.05 vs control and diabetic rats, respectively. values are mean±SEM (n=6/group)

**Table 4:** Effect of 8-week administration of nitrite on serum glucose concentration, serum insulin concentration, insulin sensitivity/resistance, and insulin secretion in rats with type 2 diabetes

**Parameters **	**Groups**		
	Control	Diabetes	Diabetes+nitrite
Serum glucose (mg/dl)	91.1±5.6	183.2±7.1*	151.4±4.6#
Serum insulin (pmol/L)	69.4±7.2	155.8±10.4*	125.2±8.5#
Islets GIIS (pmol/Islet/60 min)	0.96±0.03	0.53±0.03*	0.74±0.02#
HOMA1-IR	2.1±0.3	10.2±0.9*	6.7±0.3
HOMA2-IR	1.4±0.2	4.0±0.3*	3.0±0.2#
QUICKI	0.340±0.007	0.277±0.003*	0.298±0.005*

*, # *P*<0.05 vs control and diabetic rats, respectively

GIIS: Glucose-induced insulin secretion; HOMA1-IR: Homeostasis model assessment of IR; HOMA2-IR: Updated HOMA-IR; QUICKI: Quantitative insulin-sensitivity check index; values are mean±SEM (n=6/group)

## Conclusion

Nitrite administration in diabetic rats decreased oxidative stress in isolated pancreatic islets by suppressing oxidants and augmenting anti-oxidants. Therefore, nitrite-induced insulin secretion is at least in part due to decreased oxidative stress. These findings are relevant for potential translational nutritional-based intervention studies using nitrite. 

## Authors’ Contributions

SJ, SG, KK, and AG designed the experiments; SJ, SG, and AG performed experiments and collected data; SJ and AG discussed the results and strategy; AG supervised, directed, and managed the study; SJ, SG, KK, and AG approved the final version to be published.

## Conflicts of Interest

None.
